# Comparison of proliferative and multilineage differentiation potentials of cord matrix, cord blood, and bone marrow mesenchymal stem cells

**DOI:** 10.4103/0973-6247.59386

**Published:** 2010-01

**Authors:** Prathibha Shetty, Khushnuma Cooper, Chandra Viswanathan

**Affiliations:** *Regenerative Medicine Group, Reliance Life Sciences Pvt. Ltd., Navi Mumbai, India*

**Keywords:** Mesenchymal stem cells, bone marrow, umbilical cord blood, umbilical cord

## Abstract

**Background:**

Hematopoietic stem cells (HSCs) and mesenchymal stem cells (MSCs) are the two widely studied and characterized adult stem cells. Thus far, MSCs were obtained from the bone marrow, which is a painful procedure. Therefore, MSCs from less common sources like cord blood, adipose tissue, tooth pulp, and so on, have been the subject of research. The purpose of this study is to explore the possibility of finding MSCs from a less controversial, easy, and abundant source, such as the umbilical cord, for potential regenerative medicine applications.

**Study Design and Methods:**

Five bone marrow samples (BM), seventy cord blood units (CB), and four umbilical cord matrix (CM) samples have been used for the study. Expanded MSCs were checked for biomarker expression by flow cytometry and were also checked for their differentiation to mesodermal and ectodermal lineages.

**Results:**

MSCs could be isolated from 100% BM and CM samples, as compared to only 6% of CB samples. The fold expansion of the mesenchymal stem cells observed in CB (CB-MSCs) was distinctly higher as compared to BM (BM-MSCs) and CM (CM-MSCs). MSCs isolated from all the three sources expressed a characteristic mesenchymal phenotype of CD45 − /vWF − /CD14 − /CD31 − /CD73 + /CD105 + /SSEA4 + /CD29 + /CD44 + /HLAABC +, whereas, the HLA DR was conspicuously absent in CM-MSCs and CB-MSCs. Although osteogenic, chondrogenic, and neural differentiation was observed in MSCs from all sources, adipogenic differentiation was observed only in BM-MSCs.

**Conclusion:**

CM-MSCs are a dependable source of an unlimited number of MSCs for autologous and allogenic use in regenerative medicine.

## Introduction

Current treatments for various degenerative disorders rely on surgical interventions and drugs that modulate the system, but these have their own limitations when it comes to regeneration of damaged tissues and cells. To address this shortcoming, cell-based therapies are gaining importance. Among the different types of stem cells studied adult stem cells such as mesenchymal stem cells (MSCs) are most widely researched for their therapeutic applications.

MSCs represent a promising tool for potential therapeutic applications characterized by cell loss or cell degeneration.[1] They are characterized as undifferentiated cells, able to self-renew with a high proliferative capacity, and possess a mesodermal differentiation potential.[2] Human MSCs were first identified in postnatal bone marrow and later in a variety of other human adult tissues, including muscle, connective tissue, skin, adipose tissue, perichondrium, trabecular bone, placenta, umbilical cord blood, and umbilical cord.[3,7] This explains why many researchers have chosen to work with MSCs from the traditional and nonconventional sources.

Making MSCs available for regenerative medicine applications in adequate quantities and at the right time is a challenge. Clinical situations will demand MSCs with dependable quality and quantity to treat various disorders representated by cellular degeneration. Currently, BM represents the main source of MSCs for both experimental and clinical studies, but with limitations. Besides, bone marrow aspiration being an invasive procedure, its frequency and differentiation capacity decline with age, reducing their therapeutic potential.[4] Therefore, the search for alternative sources of MSCs is of significant value.

In this study we demonstrate the ease of isolation of MSCs from a relatively newer, unique, easily available, and noncontroversial source, which is the human umbilical cord matrix. Besides comparing the characteristics in terms of derivation, growth kinetics, phenotype, and plasticity, our study also highlights the possibilities of umbilical cord matrix mesenchymal stem cells (CM-MSCs) overcoming the limitations of BM-MSCs and CB-MSCs.

## Materials and Methods

### Bone marrow collection and processing

Five to ten milliliters of bone marrow were collected from the posterior superior iliac spine into heparinized vacutainers after a due consenting process. This was approved by the local hospital ethics committee. We included only those volunteers between the ages of 30 to 50 years. Marrow samples were transported to our laboratory at temperatures of 4-8°C, by maintaining a validated cold chain. All the processes on the sample began within 24 hours of sample collection.

MSCs were isolated and expanded as per modifications of the previously established protocols of Pittenger *et al*.[2] The heparinized BM was mixed with phosphate-buffered saline (PBS) (Invitrogen Corp., USA) and the cells were washed once at speeds standardized in the laboratory at 1600-2000 rpm, for five minutes. The washed cells were diluted with PBS and layered onto equal volumes of 1.073 g/ml Percoll (Sigma, USA) and centrifuged at 400 g for 20 minutes to obtain mononuclear cells (MNCs). The MNCs from the PBS — Percoll interface were diluted with media and plated into tissue culture flasks (Nunc, Denmark) for MSC derivation.

### Cord blood collection and processing

Human CB units from full-term, normal, and cesarean deliveries, from a heterogeneous ethnic mix were collected with the approved informed consent of the mothers. A blood bag containing a citrate phosphate dextrose adenine USP anticoagulant was used for the CB collections (Terumo Penpol Ltd., India). The units were stored at ambient temperature before processing.

The CB was processed within 24 hours of collection and depleted of red blood cells (RBCs) using high molecular weight dextran (GE Health Care Biosciences, Sweden) according to the protocol described by Tanavde *et al*.[6] RBC depletion was carried out using 3% v/v Dextran in the ratio of 1:1 with respect to the volume of blood. Leucocyte-rich plasma was collected carefully and centrifuged to obtain leucocytes. The leucocytes were layered on Histopaque® 1077 (Sigma, USA) and centrifuged at 400 g for 20 minutes.[6] MNCs were separated from the interface, washed, counted, and suspended in a MSC proliferation medium.

### Culturing of MNCs for MSC isolation from bone marrow aspirate and umbilical cord blood

Mesenchymal stem cells from CB and BM aspirates were plated in 75 cm^2^ tissue culture flasks (Nunc, Denmark), in an MSC proliferation medium. For CB, the proliferation medium consisted of DMEM/F12 (1:1) (Invitrogen Corp., USA), supplemented with 10% serum, and for bone marrow, the commercially available Mesenchymal Stem Cell Growth Media (Lonza, USA) was used. In addition the medium also contained 1-5 ng/ml fibroblast growth factor (bFGF) (Sigma, USA). The cells were seeded at a density of 1 × 10^6^ to 10 × 10^6^ cells/ml. The cultures were maintained in a 37°C incubator containing 5% CO_2_ (Forma Scientific, Germany). The cells adhered to the surface of the tissue culture flask within 24 hours and the media were replenished regularly. Upon reaching confluency the adherent cells were harvested using trypsin EDTA (Invitrogen Corp., USA). The harvested cells were replated at a low density of < 5000 cells/cm^2^ for all subsequent passages. The harvested cells were also analyzed for the expression of CD73, CD105, CD45, SSEA4, HLADR, HLAABC, CD14, CD31, CD44, CD29, and vWF markers, by flow cytometry.

### Umbilical cord collection

Umbilical cords were obtained from the maternity hospitals after normal or cesarean deliveries. The cords were collected after obtaining the consent of the mother. Samples were collected into tubes containing DMEM (Invitrogen Corp., USA) supplemented with antibiotics, and were processed within 24 hours of collection.

### Processing and culturing of mesenchymal stem cells from umbilical cord

The umbilical cords were washed with PBS containing antibiotics, cut open longitudinally, and the blood vessels removed. The cord matrix was then serially cut in a cross-sectional manner and four to five explants of the matrix, ranging from 1-2 cm, were placed in 100 mm tissue culture dishes with 2-3 ml culture medium. The dishes were left undisturbed for 3-4 days, after which fresh cell culture media (MSC proliferation media) was added to the dishes.

Adherent cells were allowed to expand with regular media changes. The cells were harvested at 80-90% confluence using trypsin EDTA and replated into 75 cm^2^ tissue culture flasks at a density of 5 × 10^3^ cells/cm^2^ in cell culture media.

### Immunophenotypying of cultured mesenchymal stem cells isolated from bone marrow aspirate, umbilical cord blood, and umbilical cord

Immunophenotyping of the expanded MSCs was done using flow cytometry. The adherent cells were washed with PBS and detached by incubating with trypsin EDTA for 5 minutes at 37°C. The harvested cells were washed using staining buffer containing 4% FBS and 0.1% azide in PBS. After harvesting the adherent cells, the cell count was taken and approximately 0.05-0.1 × 10^6^ cells per tube were used for cell surface antigen expression studies. The cells were incubated with CD45 PerCP (BD biosciences, USA), CD73 PE (BD Pharmingen, USA), CD105 PE (Caltag laboratories, USA), SSEA4 PE (R and D systems, USA), HLADR PE (BD biosciences, USA), HLAABC PE (BD Pharmingen, USA), CD14 PE (BD Pharmingen, USA), CD31 PE (BD Pharmingen, USA), CD29 PE (BD Pharmingen, USA), CD44 PE (BD Pharmingen, USA), and purified vWF (BD Pharmingen, USA), using the standard techniques.[8] Appropriate isotype controls from BD Pharmingen, USA were used. Goat anti-mouse FITC (BD biosciences, USA) was used as secondary antibody for vWF antibody.

These cells were acquired on a FACS Calibur flow cytometer (Beckton Dickinson, USA) equipped with a 488 nm Argon Laser. Approximately 10,000 events were acquired and analyzed using the Cell Quest software. For viability determination, the cells were stained with 7-Amino Actinomycin D (7-AAD), (BD Pharmingen, USA), and acquired on the flow cytometer. Dead cells took up the fluorescent stain, while the live cells excluded this stain. Viability and surface antigen studies were done at every passage.

### Differentiation Studies

#### Osteogenic potential

Mesenchymal stem cells isolated from all the three sources namely BM, CB, and CM were expanded in the proliferation media. For osteogenic differentiation the expanded cells were plated in an eight-well chamber slide with a count of 3000-10,000 cells per well. After plating the cells in proliferation medium for 24 hours, osteogenesis was induced by replacing the proliferation medium with the commercially available osteogenic induction medium (Lonza, USA). The differentiation medium was changed every two to three days. After 21 days of differentiation the cells were checked for calcium deposition by Von Kossa technique.[9]

#### Chondrogenic potential

For chondrogenic differentiation the expanded cells from all the three sources were harvested and differentiated into chondrocytes in pellet cultures at a density of 0.25-0.5 × 10^6^ in a polypropylene tube containing commercially available chondrogenic differentiation medium (Lonza, USA) to which 10 ng/ml TGFβ3 (Sigma, USA) was added as an inducing agent. The tubes were incubated at 37°C in a 5% CO_2_ incubator. The cells grew as pellets, which were fed with fresh chondrogenic medium every two to three days. Chondrogenic pellets were harvested after 14 to 28 days in the culture.

The harvested chondrogenic pellets were fixed with 10% formalin and paraffin embedded for histological processing. Thin sections, approximately 4-10 μ, were then stained with Alcian Blue as per the protocol described by Lee *et al*.[10] Sections were also stained with Safranin O.

#### Adipogenic potential

For adipogenic differentiation, the adipogenic induction medium was prepared with DMEM (High Glucose) containing 10% serum, dexamethasone (1 μM), Insulin (10 μg/ml), IBMX (0.5 mM), and Indomethacin (60 μM). The adipogenic maintenance medium consisted of DMEM (High Glucose) containing 10% serum and insulin (10 μg/ml). All inducing agents were procured from Sigma, USA.

MSCs from all the three sources were plated in an eight-well chamber slide at 5,000-10,000 cells/well in the MSC proliferation medium and incubated in a 37°C incubator with 5% CO_2_. Once the cells reached confluence they were cultured with the adipogenic induction medium for three days followed by adipogenic maintenance medium for three days. Three cycles of induction/maintenance was carried out for optimal adipogenic differentiation.

MSC monolayers were cultured for 18 days. After 18 days the spent media was discarded and the cells were washed with PBS and stained with Oil Red O for 15 minutes on a shaker at RT, rinsed with distilled water, and mounted with mounting media.

#### Neural differentiation

For neural differentiation, MSCs from all the three sources were expanded in the presence of MSC proliferation medium. These expanded cells were plated in an eight-well chamber slide (BD, Falcon) at a density of 5,000-10,000 cells per well. The slides were incubated at 37°C in a 5% CO_2_ incubator, to allow the cells to become confluent. At this stage the MSC proliferation medium was replaced with neural proliferation medium consisting of DMEM/ F12 (1:1), supplemented with 10% serum, 1 ng/ml bFGF (Sigma, USA), 50 ng/ml Human noggin (Peprotech, Germany), 2% B27 (Sigma, USA), and 100 ng/ml nerve growth factor (Sigma, USA). The cells were maintained in this medium for a week. After one week of incubation in a neural proliferation medium, the cells were induced for differentiation in a neural induction medium that consisted of a neural proliferation medium with 200 μM Butylated Hydroxy Anisole (BHA) (Sigma, USA) as an inducer. The cells were induced for five hours, after which the cells were fixed with 4% paraformaldehyde (Polysciences Inc., USA). The induced cells were checked for expression of neural markers such as Nestin, β tubulin (BT), Neurofilament, Tyrosine Hydroxylase (TH), NeuN, and Glial fibrillary acidic protein (GFAP). All the antibodies were procured from Chemicon (Invitrogen Corp., USA).

### Immunocytochemistry of differentiated BM-MSCs, CB-MSCs and CM-MSCs

The cells fixed with 4% paraformaldehyde were washed with 1× PBS and stained for the respective neural markers. The cells were first stained with the primary antibody Nestin (1:100), BT (1:100), TH (1:100), Neurofilament (1:100), NeuN (1:100), and GFAP (1:100).The primary antibody was detected using secondary goat anti-mouse Alexa 488 and Alexa 568 (Molecular Probes, USA). Only the secondary antibody was kept as a control, to check for its non-specific binding with the cells. The stained cells were observed under an inverted fluorescent microscope (Nikon, USA).

### Soft agar assay

To confirm that MSCs from all the three sources did not possess malignant properties, *in vitro* colony formation assay in a soft-agar medium was performed.

1 × 10^4^ MSCs were mixed with 0.3% agar containing DMEM media and overlayed on 0.6% agar containing DMEM media in a 35 mm tissue culture petridish (Nunc, Denmark). Approximately 300 μl of media was added on top of the agar to keep the surface moist. This addition of media was done every three days. The cells were observed for three weeks for the formation of colonies. The B16 melanoma cell line was used as a positive control.

## Results

A total of five BM aspirates, 70 CB units, and four CMs were processed for MSC isolation. Of 70 cord blood units processed, MSCs could be derived from only four samples, the remaining 66 samples were pursued for 15 days and later discarded due to the absence of MSCs. All five bone marrow samples and four umbilical cord samples showed a good presence of MSCs. Once isolated the cells could be expanded and differentiated *in vitro*. The isolated and expanded cells could be cryopreserved and expanded as and when needed for further studies.

### Cell morphology

**Figure 1:**
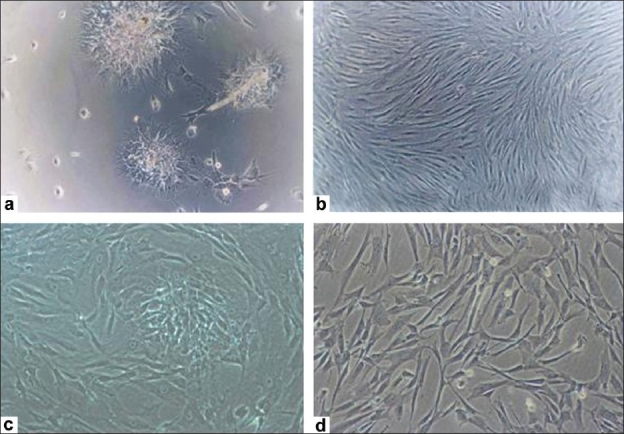
Morphology of the mesenchymal stem cells from different adult sources. (a) BMMSC formed colonies on adhering to the surface; (b) And grew as a monolayer of cells; (c) CBMSCs grew a monolayer of fibroblast like cells; (d) CM-MSC formed a monolayer of cells by day 25

After the initial plating of mononuclear cells from the BM aspirate, few fibroblast-like cells attached to the surface of the flasks within three to four days and grew as single cells. These single cells formed distinct MSC colonies after 7-10 days of culture [Figure 1a]. This was a very consistent and unique characteristic observed in case of the BM-MSCs. These colonies expanded in number and formed a homogenous monolayer of adherent fibroblast-like cells [Figure 1b].

The MSCs isolated from CB grew as adherent fibroblast-like cells and formed a monolayer of spindle-shaped cells after 15 days in the culture [Figure 1c]. However, those from the cord matrix, although they formed a monolayer, took slightly longer, as much as three to four weeks, to become confluent [Figure 1d].

### Growth kinetics

**Figure 2:**
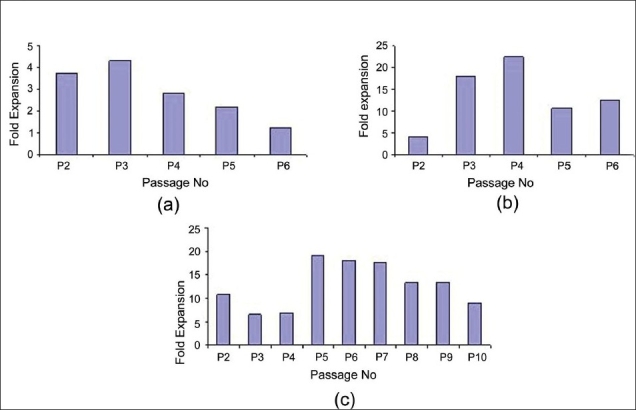
Growth kinetics of the Mesenchymal stem cells from different sources. (a) Fold expansion of MSCs isolated from BM ranged from 1.2 to 4.3 fold; (b) CBMSC showed an expansion of 4 to 22.5 fold and; (c) CM-MSCs showed an expansion of 6.6 to 19.1 fold. Average time between two passages ranged between 7-8 days and was consistent for all the different types of MSCs

Bone marrow-mesenchymal stem cells showed exponential growth throughout the study period. The primary cultures took about 15 to 20 days and the cell count ranged from 0.5 × 10^6^ to 10 × 10^6^. The growth was consistent with an average fold expansion of 1.2-4.3 folds. The growth of BM-MSC showed a decline, which practically stopped at the end of the sixth passage [Figure 2a]. The expansion of CB-MSCs was studied up to six passages and at every passage the expanded cells showed an average of it has be 4-22 fold expansion. The MSC count obtained at the end of each passage ranged from 0.3 × 10^6^ to 9.0 × 10^6^ cells [Figure 2b]. CM-MSCs were also expanded in culture by repeated harvesting and replating of cells every seven to eight days, up to passage 10. The mean number of MSCs harvested after passage 10 was 7.9 × 10^16^. From the explant stage (P0) to passage P10, the CM-MSCs showed an 11-fold expansion. [Figure 2c]. We believe that CM-MSCs can be cultured for several more passages and further studied for their potentials.

Furthermore, the mean population doubling time of the MSCs from different sources was also calculated. It was observed that the mean population doubling time of CM-MSCs and CB-MSCs was 35 hours and 44 hours respectively. BM-MSCs showed a mean doubling time of 85 hours, which was the highest in comparison to the MSCs derived from the other two sources.

### Biomarker expression by flow cytometry

**Figure 3:**
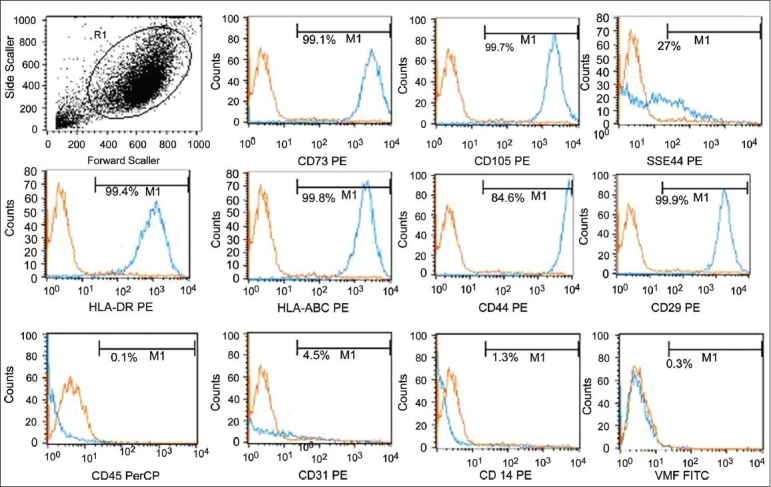
Immunophenotyping of the expanded BM-MSCs by flow cytometry. The expanded BM MSCs were negative for haematopoeitc marker and strongly expressed the mesenchymal markers. The phenotype expressed by the expanded cells was CD73 + /CD105 + /CD29 + /CD44 + /SSEA4 + /HLA ABC + /HLA DR + /CD45 − / CD14 − /CD31 − /vWF −

**Figure 4:**
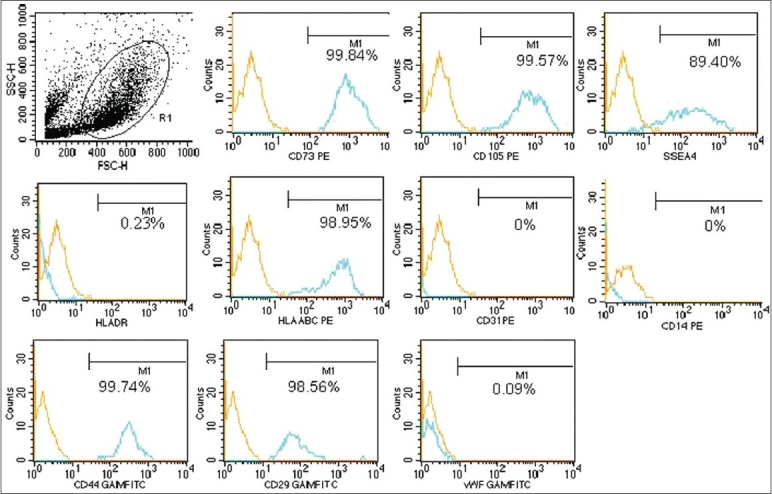
Immunophenotyping of the expanded CB-MSCs by flow cytometry. The expanded CBMSCs were negative for haematopoeitc marker and strongly expressed the mesenchymal markers. The phenotype expressed by the expanded cells was CD73 + /CD105 + /CD29 + /CD44 + /SSEA4 + /HLA ABC + /HLA DR − /CD45−/CD14 − /CD31−/vWF −

**Figure 5:**
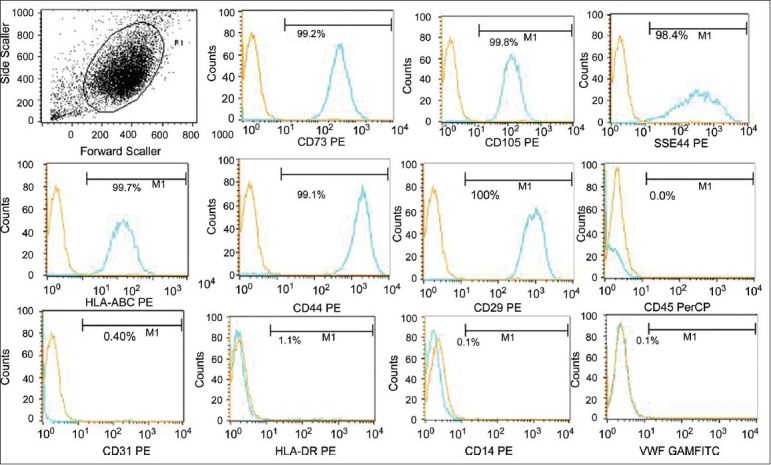
Immunophenotyping of the expanded CM-MSCs by flow cytometry. The expanded CM-MSCs were negative for haematopoeitc marker and strongly expressed the mesenchymal markers. The phenotype expressed by the expanded cells was CD73 + /CD105 + /CD29 + /CD44 + /SSEA4 + /HLA ABC + /HLA DR-/CD45-/CD14-/CD31-/vWF-

Mesenchymal stem cells from all the three sources were checked for biomarker expression by flow cytometry. These cells were negative for the hematopoietic marker CD45, endothelial markers vWF, and CD31, monocyte marker CD14. MSCs isolated from the BM showed expression of HLA DR, while those isolated from the umbilical cord blood and cord matrix were negative for HLA DR expression. MSCs strongly expressed surface proteins CD73, CD105, CD29, CD44, HLAABC, and weak-to-moderate expression of SSEA-4. Over 90% MSC expressed the phenotype of CD45 − / vWF − /CD14 − /HLADR ± /CD31 − /CD73 + /CD105 + /SSEA4 + /CD29 + /CD44 + /HLAABC + [Figure 3–5].

### Differentiation potential of mesenchymal stem cells from bone marrow samples, UCB, UC

#### 

##### Osteogenic differentiation

**Figure 6:**
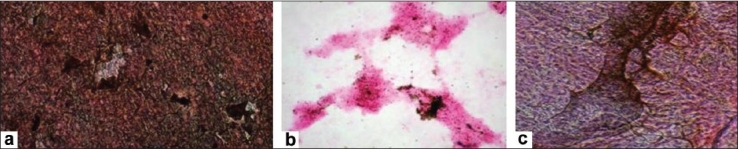
Differentiation of mesenchymal stem cells to osteocytes. Von kossa staining of (a) BM-MSC; (b) CB-MSC; (c) CM-MSC

A qualitative examination to look for osteogenic differentiation showed the presence of mineralized nodules under the microscope (Zeiss Axiovert 25) after staining with the Von Kossa protocol. Cells cultured for 21 days were checked for calcium deposition, which appeared as a brown precipitate [Figure 6]. Qualitatively it appeared that BM-MSCs showed increased mineralization as compared to CB and CM-MSCs. Uninduced MSCs served as a negative control.

### Chondrogenic differentiation

Upon chondrogenic differentiation the MSCs formed a pellet within one day and the size increased over a period of two to three weeks. After two to three weeks of culture a spheroid cell mass was formed, which was fixed and paraffin embedded. Thin sections were made, which were stained using Alcian blue and Safranin O. The sections stained positive with both Alcian blue [Figure 7a] and Safranin O [Figure 7b] indicating the presence of a cartilage matrix.

**Figure 7a:**
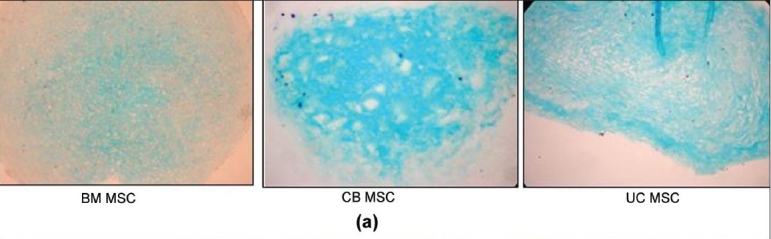
(a) Differentiation of mesenchymal stem cells to chondrocytes. Immunohistochemical staining of pellet cultures from different sources for detection of proteoglycans. (a) Alcian blue staining

**Figure 7b:**
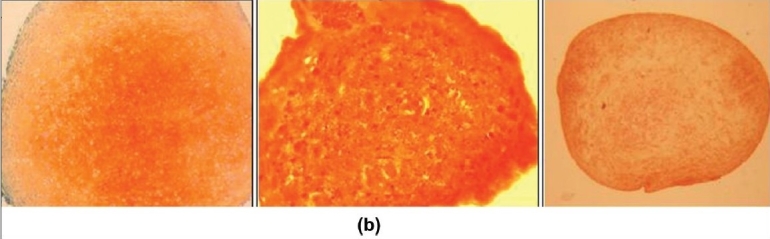
Differentiation of mesenchymal stem cells to chondrocytes. Immunohistochemical staining of pellet cultures from different sources for detection of proteoglycans. (b) Safranin O staining

### Adipogenic differentiation

**Figure 8:**
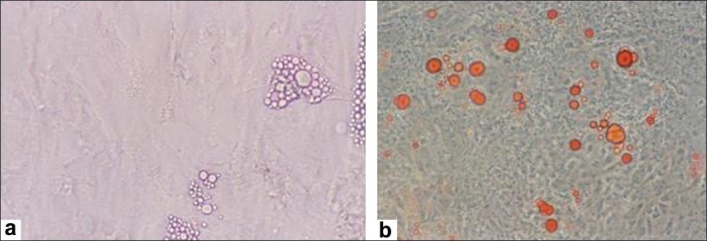
Differentiation of mesenchymal stem cells to adipocytes. (a) Adipogenesis of BMMSCs was detected by the presence of lipid vacuoles; (b) which stained positive with oil red O

When the MSCs from all the three sources were exposed to the adipogenic medium, the BM-MSCs showed presence of lipid vacuoles, which were detected by phase contrast microscopy (Zeiss Axiovert 25) [Figure 8a]. Adipogenesis was confirmed by the presence of these vacuoles staining positive with Oil Red O [Figure 8b]. Both CB-MSCs and CM-MSCs did not show any lipid vacuoles on treating with the adipogenic media in several experiments performed.

### Neural differentiation

#### Immunoflourescence

**Figure 9:**
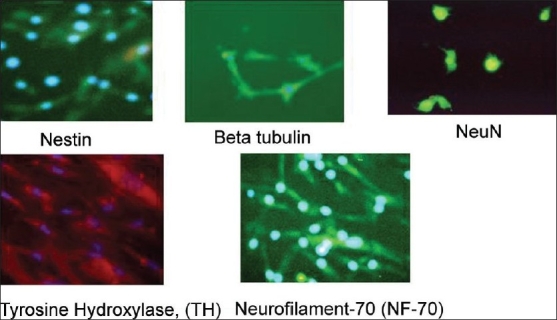
Neuronal differentiation of expanded BM-MSCs. Differentiated BM-MSCs expressed neural specific markers such nestin, beta tubulin, neuN, TH, NF-70

**Figure 10:**
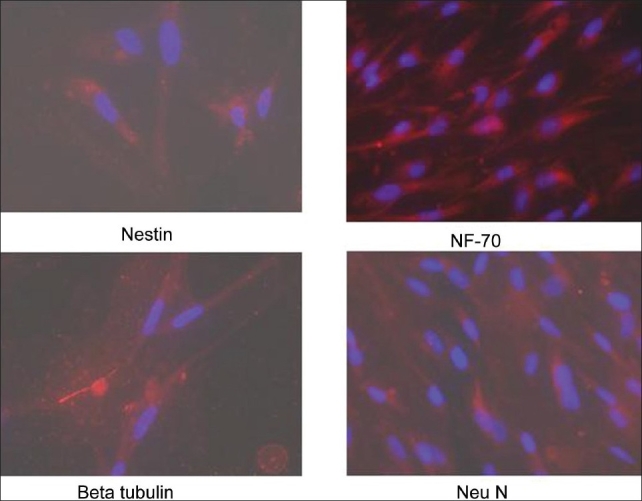
Neuronal differentiation of expanded CB-MSCs. Differentiated CBMSCs expressed neural specific markers such nestin, beta tubulin, NF-70, neuN

**Figure 11:**
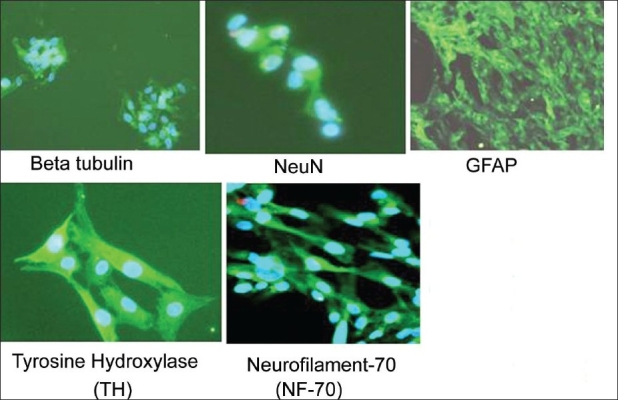
Neuronal differentiation of the expanded CM-MSCs. Differentiated CM-MSCs expressed neural specific markers such as beta tubulin, NeuN, GFAP, TH, NF-70

Morphologically and phenotypically characterized MSCs derived from three different sources were subjected to a neural proliferation medium. Upon induction with BHA they expressed neural markers, which were checked by immunoflouresence. These induced cells were found to be positive for neural markers such as Nestin, GFAP, NeuN, BT, Neurofilament (NF-70), and TH [Figures 9–11].

### Soft agar assay

**Figure 12:**
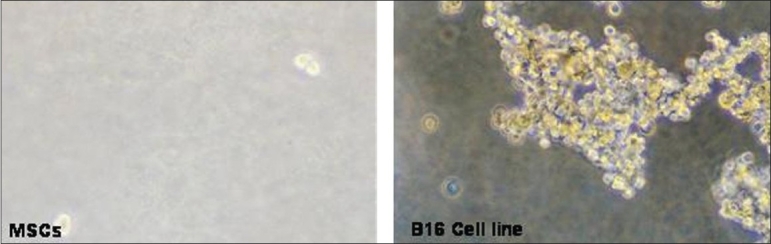
Soft agar assay. MSCs from all the three sources did not form colonies in soft agar assay confirming that they do not possess tumorogenic properties

B16 melanoma cells started growing as colonies after five days. By the end of 14 days big colonies had formed. However, MSCs from all the three sources did not form colonies till the end of 21 days [Figure 12].

## Discussion

Mesenchymal stem cells as a choice for cell-based therapies in regenerative medicine are becoming very clear.[17] The increasing amount of literature indicating its usefulness in clinical applications is very encouraging.[18,19] As we will soon move from autologous therapies to allogeneic therapies, the challenge will be the availability of a non-controversial supply of starting material, in adequate numbers, to address the ever-increasing number of patients the world over, who will look forward to this mode of therapy.

In this study we have successfully isolated, expanded, and compared the MSCs from two sources such as CB and CM with BM, which is a well-studied and accessible source of MSCs. The MSCs from three different sources have been compared for their common cell surface epitopes and multilineage differentiation potentials to mesodermal and ectodermal lineages.[11]

MSCs could be easily isolated from all the five BM and four CM samples processed, as compared to CB. Of the 70 CB samples processed, only four samples, that is, 6% of the samples could yield MSCs, indicating a low frequency of MSCs in CB as reported by various groups.[12,5] This percentage of successful isolation of MSCs from CB is lower than observed by others (34-63% of UCB samples).[5,16] In sharp contrast, 100% of the CM and BM samples processed by us yielded MSCs.

Despite the low frequency of CB-MSCs, its expansion potential was the highest, as compared to the MSCs derived from other tissue sources studied by us. The presence of MSCs, in all units of CB that were processed, was independent of age, birth weight, volume, gravida, and sex of the baby. Thus, not every cord blood unit can act as a source of MSCs for either autologous or allogenic use. Therefore, the present level of understanding, based on our research of more than 70 units, necessitates that one may need to screen several cord blood units to ascertain the presence of MSCs in CB.

Cord blood - mesenchymal stem cells initially showed lower fold expansion at passage two, which increased in the third and fourth passage and later declined and remained steady after the fifth passage. CM-MSCs showed a higher fold expansion at passage two, which reduced in the third and fourth passage and later increased up to passage 10, where they remained consistent. It was observed that the BM-MSCs showed a decline in the fold expansion on subsequent passaging.

MSCs from all the three sources exhibited typical MSC characteristics: A fibroblast-like morphology, expressed classic MSC marker proteins, but lacked hematopoietic and endothelial markers, except for HLA DR expression, which was observed only in BM-MSCs.

Mesodermal lineage potentials among these various MSCs showed a marked difference in their adipocyte differentiation capability. CB-MSCs and CM-MSCs did not differentiate into adipocytes in comparison to BM-MSC, which showed a good lipid accumulation upon exposure to the adipogenic medium. Similar observations were reported by Chang *et al*.[13] In contrast to this, MSCs from all the three sources differentiated into both osteogenic and chondrogenic lineages. Under neurogenic conditions, MSCs from all the three sources also differentiated into neural and glial cells, as demonstrated by the presence of specific neural markers. This neuronal differentiation ability of the MSCs derived from all the three sources would be very useful for their applications in the treatment of various neurological disorders.

Bone marrow - mesenchymal stem cells have been used in several clinical trials in various domains, such as, cardiology, neurology, and so on, with its limitations in the autologous settings, as it represents 0.01-0.001% of the nucleated cells in the adult human marrow.[14,15,20] Allogenic BM-MSCs are also under research and in an advance phase of clinical trial for its application in cartilage injury and Crohn's disease. Hence, to overcome this limitation there was a need to search for an alternative source, which was easily available, noncontroversial, and could generate a higher number of MSCs. CB and CM could successfully replace BM as a source for MSCs. There are many reports of successful isolation and differentiation of MSCs from different sources.[11]

Our study thus shows that CM can address the issue of availability, as it is an abundant and easily available source. MSCs can be easily derived from CMs, which have high expansion potential, as seen from our results. As these MSCs do not express MHC class II antigens, they can, along with their immunomodulatory mechanisms, address several immunological issues in a clinical set up. In the recent past, MSCs have been used for CD34 expansion and GVHD prevention too.[12]

Thus, from our present study we can conclude that CM-MSCs are very versatile, dependable, and can be useful for various regenerative medicine applications.
